# Grail is involved in adipocyte differentiation and diet-induced obesity

**DOI:** 10.1038/s41419-018-0596-8

**Published:** 2018-05-09

**Authors:** Peiyao Liu, Poshiuan Hsieh, Huitsu Lin, Tejung Liu, Hsuehling Wu, Chengcheung Chen, Yingchuan Chen

**Affiliations:** 1 Department of Physiology & Biophysics, National Defense Medical Center, Taipei, Taiwan 114 Republic of China; 2 Institute of Preventive Medicine, National Defense Medical Center, New Taipei City, Taiwan 114 Republic of China; 3 Department of Physical Medicine and Rehabilitation, Tri-Service General Hospital, Taipei, Taiwan 114 Republic of China; 4 Department of Physical Medicine and Rehabilitation, School of Medicine, National Defense Medical Center, Taipei, Taiwan 114 Republic of China; 5 Department of Physical Medicine and Rehabilitation, Taoyuan Armed Force General Hospital, Taoyuan, Taiwan 114 Republic of China

## Abstract

Grail is a crucial regulator of various biological processes, including the development of T-cell anergy, antiviral innate immune response, and cancer. However, the role of Grail in adipogenesis and obesity remains unclear. Here, we demonstrated that Grail knockdown in vitro leads to a decrease in PPARγ expression, resulting in adipogenesis inhibition. However, Grail overexpression induced the same effects. Grail was shown to interact with PPARγ, targeting it for degradation and modulating its adipogenic activity. PPARγ expression was shown to be considerably reduced in Grail knockout (KO) mice fed normal diet or high-fat diet (HFD). The administration of both normal diet or HFD to Grail KO mice led to lower adipose mass and body weight than those in the wild-type mice. HFD-fed Grail KO mice had improved glucose and insulin tolerance. Taken together, our results indicate that Grail plays a pivotal role in adipogenesis and diet-induced obesity by regulating PPARγ activity.

## Introduction

Obesity is caused by an increase in the energy intake and a decrease in energy expenditure, and it represents a major risk factor for the development of type 2 diabetes and cardiovascular disease^1^. Obesity is characterized by the adipose tissue expansion owing to the increase in adipocyte number (hyperplasia) and size (hypertrophy)^2^. Adipose tissue was shown to express and secrete a variety of metabolites, hormones, and cytokines that have been implicated in the development of metabolic syndrome^3^. However, the underlying molecular mechanisms remain unclear.

Adipocytes express many pro-inflammatory cytokines that induce adipose tissue inflammation and insulin resistance, including tumor necrosis factor (TNF)-α, interleukin (IL)-6, IL-1β, and monocyte chemoattractant protein-1 (MCP-1)^4^.

Adipogenesis is defined as the differentiation of mature adipocytes from mesenchymal stem cells or preadipocyte precursor cells, and it is controlled by the sequential activation of many transcription factors^5,6^. During the early stage of differentiation, C/EBPβ and C/EBPδ expression is induced, and they subsequently upregulate the expression of two main adipogenic regulators, PPARγ and C/EBPα^7^, which act cooperatively to induce the expression of adipogenesis-related factors, completing the differentiation process^8,9^.

Grail is a type I transmembrane protein in the transferrin-recycling endocytic pathway that has a crucial role in the induction of anergy^10^. Grail forms a ternary complex with Otub-1 and USP8, regulating T-cell anergy^11,12^. In addition, Rho guanine dissociation inhibitor (RhoGDI) associates with Grail, thus mediating T-cell activation by increasing the stability of RhoGDI. RhoGDI inhibits RhoA GTPase activity, which has been shown to regulate cytoskeletal organization and IL-2 expression^13^. Moreover, we previously reported that Grail is a p53-interacting protein and that it, similar to mdm2, forms a negative feedback loop with p53^14^. These studies showed that Grail has multiple biological functions in addition to the induction of anergy. However, its role adipogenesis and obesity regulation has not been elucidated.

Here, we aimed to determine the roles of Grail in the regulation of adipogenesis and obesity in vitro and in vivo.

## Results

### Induction of Grail expression during adipogenesis

**Fig. 1:**
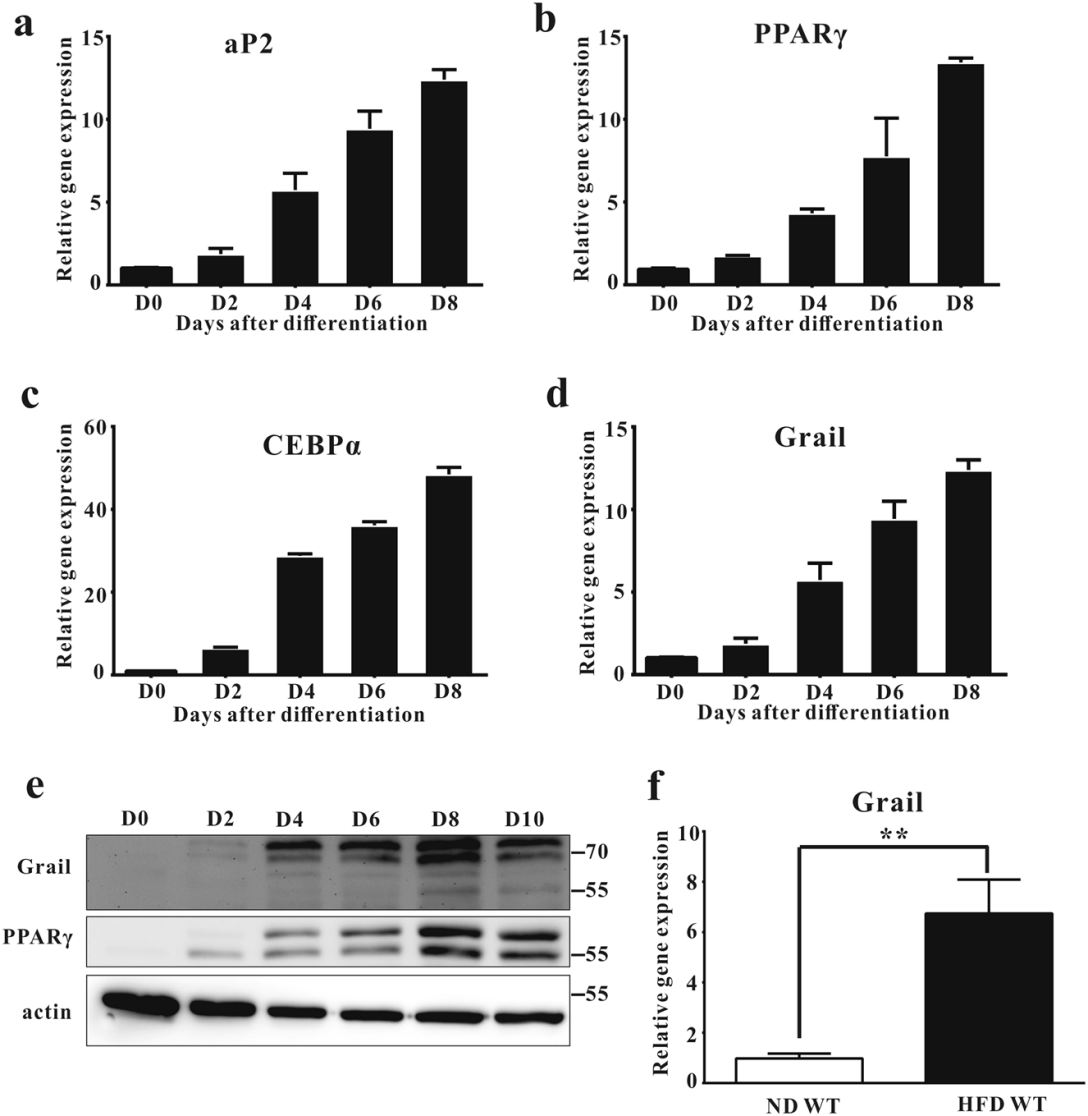
Grail expression is upregulated during adipocyte differentiation. **a**–**e** Grail expression in 3T3-L1 cells was determined using q-PCR and immunoblotting. PPARγ, C/EBPα, and aP2 were used as adipogenic markers. **f** Grail mRNA expression in the adipose tissue of HFD and ND-fed mice

We previously showed that Grail has a crucial role in the apoptosis and cell cycle progression^14^, but its role of in adipogenesis remained unknown. Therefore, we investigated the expression of Grail during adipogenic differentiation. Similar to that of the adipocyte markers PPARγ, C/EBPα, and aP2, Grail expression was shown to be induced during 3T3-L1 adipocyte differentiation (Fig. 1a–e). The same expression pattern was observed in 3T3-F442A and human mesenchymal stem cells during adipogenic differentiation (Supplementary Fig. 1). Animal studies showed that Grail expression was considerably higher in adipose tissue isolated from high-fat diet (HFD)-fed obese mice than in that obtained from the lean controls (Fig. 1f).

### Grail knockdown inhibits adipocyte differentiation

**Fig. 2:**
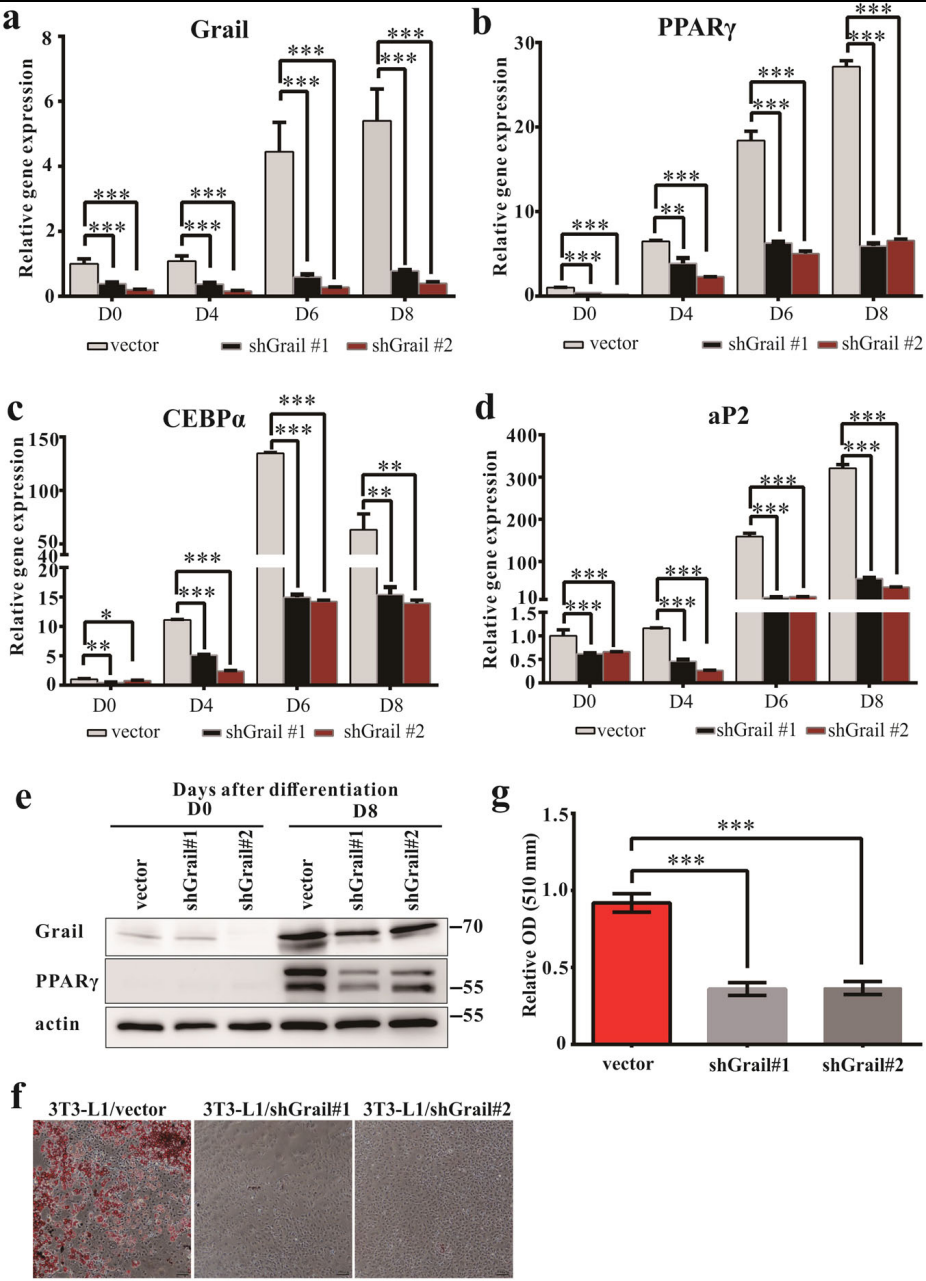
Inhibition of Grail prevents adipocyte differentiation. **a**–**d** Grail, PPARγ, C/EBPα, and aP2 mRNA expression levels in 3T3-L1/vector and 3T3-L1/shGrail cells at the indicated post-induction times points. **e** Grail and PPARγ protein levels in 3T3-L1/vector and 3T3-L1/shGrail cells at the indicated time points following the induction of differentiation. **f**–**g** Photomicrographs of differentiated Oil Red O-stained 3T3-L1/vector and 3T3-L1/shGrail cells after 8 days of differentiation. The data are presented as mean values ± SD obtained in three independent experiments. ^*^P < 0.05; ^**^P < 0.01; ^***^P < 0.001, Student’s *t-*test

We further characterized the relationship between Grail and adipogenic differentiation, by inhibiting Grail expression in 3T3-L1 (3T3-L1/short hairpin (sh)Grail) cells, and confirmed the downregulation of Grail expression (Fig. 2a, e). Furthermore, we measured the expression of adipogenesis-related genes at several time points during adipocyte differentiation. The expression levels of PPARγ, C/EBPα, and aP2 were decreased in 3T3-L1/shGrail cells compared with those determine in the controls (Fig. 2b–e). Oil Red O staining was used to analyze cell differentiation, and the results showed that Grail knockdown induced a decrease in differentiation capacity, compared with that observed in the controls (Fig. 2f, g).

### Grail is necessary for adipogenesis

**Fig. 3:**
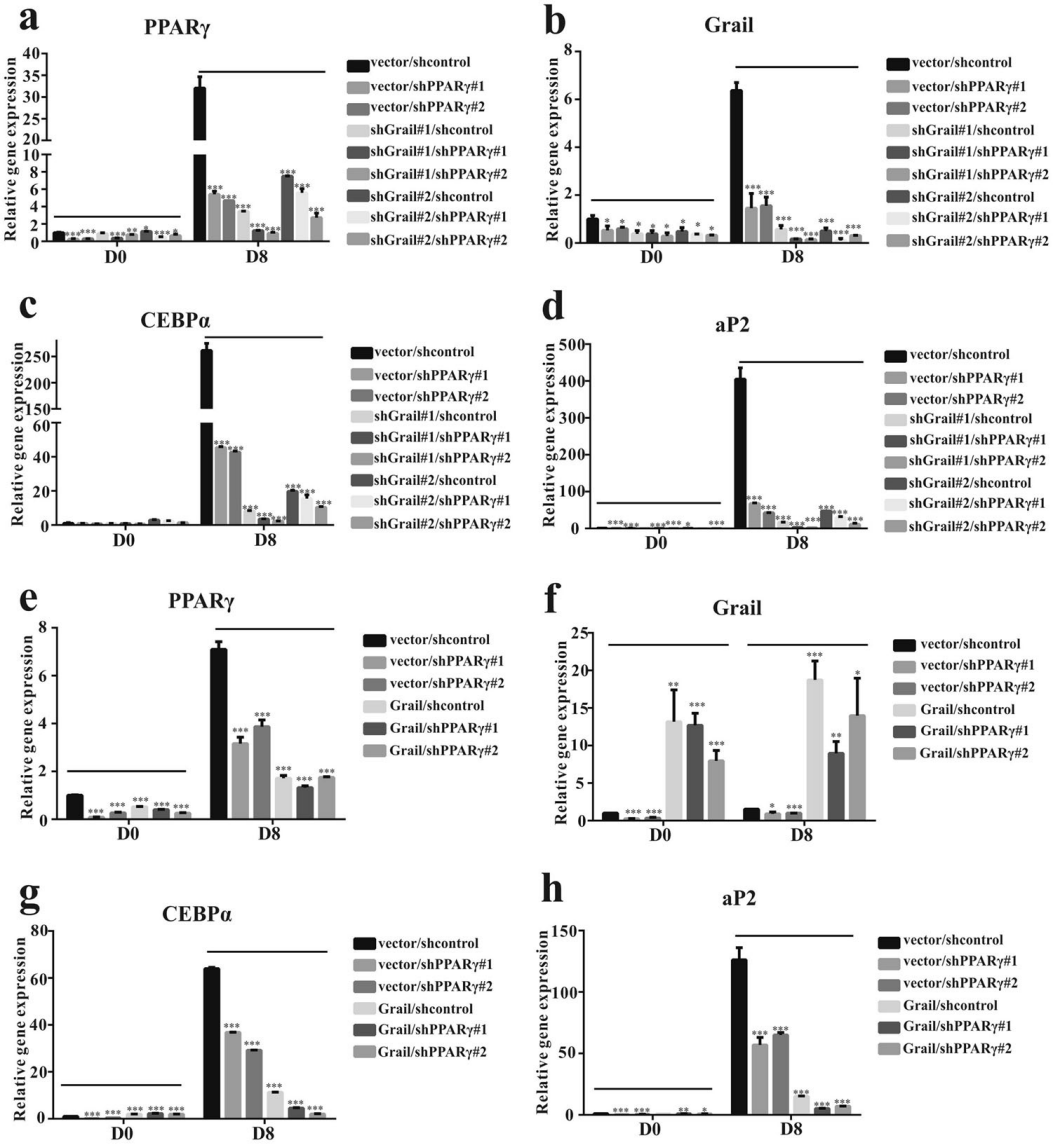
Grail is required for adipogenesis. **a**–**h** Grail, PPARγ, C/EBPα, and aP2 mRNA expression levels in 3T3-L1/vector/shPPARγ and 3T3-L1/shGrail/shPPARγ and 3T3-L1/Grail/shPPARγ cells at the indicated time points after differentiation

A previous study showed that PPARγ is the master regulator of adipogenesis^7^. Therefore, we analyzed the relationship between Grail and PPARγ expression during adipocyte differentiation. Adeno-associated viral (AAV) particles, carrying two independent PPARγ shRNAs, were used to infect 3T3-L1/vector and 3T3-L1/shGrail cells, and the expression levels of adipogenic genes were examined. PPARγ expression was confirmed to be downregulated (Fig. 3a), followed by a decrease in the expression levels of PPARγ, C/EBPα, aP2, and Grail in the 3T3-L1/vector/shPPARγ and 3T3-L1/shGrail/shPPARγ cells, compared with those in the controls (Fig. 3a–d). Similarly, PPARγ knockdown in the 3T3-L1/vector and 3T3-L1/Grail cells led to the reduction in PPARγ, C/EBPα, and aP2 expression levels (Fig. 3e–h). Grail expression decreased as well in the PPARγ-silenced cells (Fig. 3b, f).

### Grail overexpression prevents adipogenesis

**Fig. 4:**
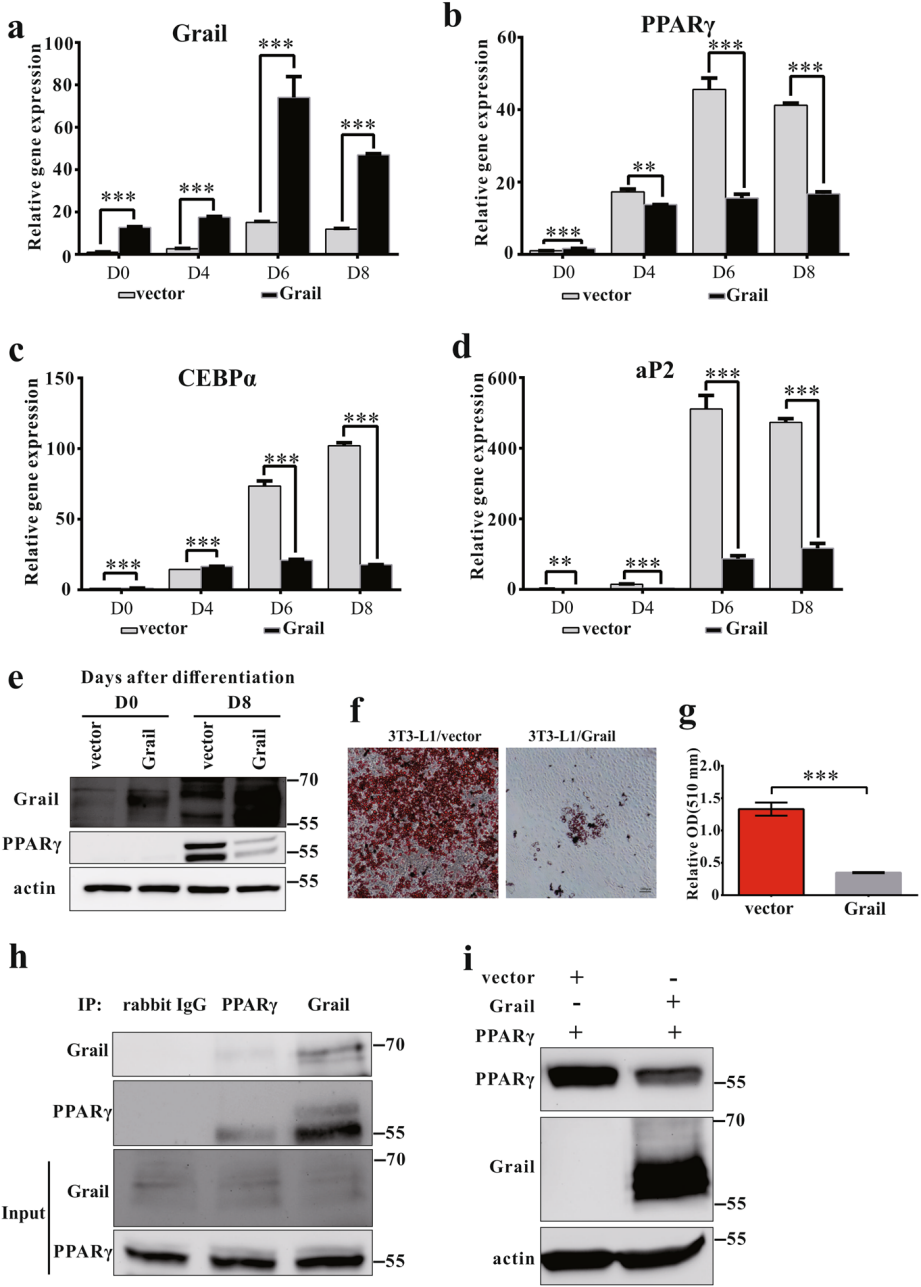
Grail interacts with PPARγ, targeting it for degradation. **a**–**d** Grail, PPARγ, C/EBPα, and aP2 mRNA levels in 3T3-L1/vector and 3T3-L1/Grail cells at the indicated time points after differentiation. **e** Grail and PPARγ protein levels in 3T3-L1/vector and 3T3-L1/Grail cells at the indicated time points. **f**–**g** Oil Red O staining of 3T3-L1/vector and 3T3-L1/Grail cells after 8 days of differentiation. **h** Endogenous Grail interacts with endogenous PPARγ. Extracts from differentiated 3T3-L1 cells were prepared, immunoprecipitated with anti-Grail, anti-PPARγ, or rabbit anti-IgG antibodies, and anti-Grail and anti-PPARγ antibodies were used for the analysis. **i** Effects of Grail overexpression on PPARγ degradation. HEK293 cells were transfected with 0.5 μg of vector or Grail in the presence of 0.5 μg of pcDNA-flag-PPARγ. The cells were then harvested and subjected to western blotting. The data are presented as mean ± SD obtained in three independent experiments. ^*^P < 0.05; ^**^P < 0.01; ^***^P < 0.001, Student’s *t-*test

We determined the effects of Grail overexpression on the adipogenic differentiation of 3T3-L1 cells. Full-length Grail cDNAs were stably overexpressed in 3T3-L1 cells using the retroviral transduction, and Grail levels were determined at mRNA and protein levels (Fig. 4a, e). In these cells, the expression of PPAR-γ, CEBP-α, and aP2 was shown to be significantly decreased than that in the controls (Fig. 4b–d). Oil Red O staining results showed extensive adipocyte differentiation with larger lipid droplets in the 3T3-L1/vector cells than that in the 3T3-L1/Grail-overexpressing cells (Fig. 4f, g).

### Grail regulates PPARγ protein levels

As Grail has been characterized as an E3 ligase, we analyzed whether Grail interacts with PPARγ and targets it for degradation, by using a co-immunoprecipitation assay with 3T3-L1 cells. A reciprocal interaction between Grail and PPAR-γ was observed in the differentiated 3T3-L1 cells (Fig. 4h), and we showed that Grail overexpression led to a decreases in PPARγ protein levels (Fig. 4i and Supplementary Fig. 5).

### Grail knockout (KO) mice are protected against diet-induced obesity

**Fig. 5:**
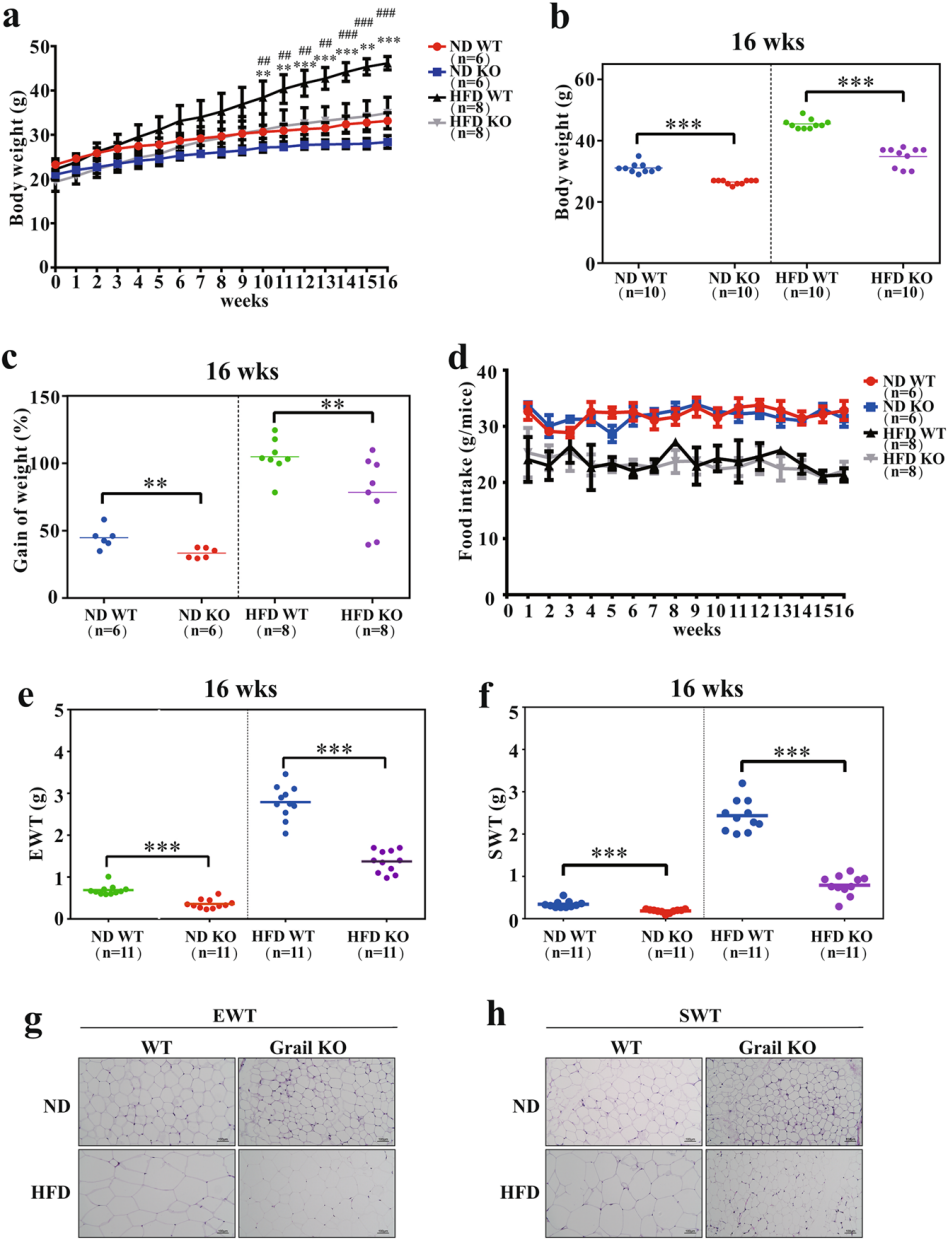
Diet-induced obesity is limited in the Grail KO mice. **a** Six-week-old WT and Grail KO mice were fed ND or HFD for 16 weeks, and their weights were determined. **b**–**c** Body weight and weight gain of the ND- or HFD-fed WT and Grail KO mice at 16 weeks. **d** Food intake of the ND- and HFD-fed WT and Grail KO mice. **e**–**h** Weight and H&E-stained EWT and SWT sections obtained from the WT and Grail KO mice fed for 16 weeks with ND or a HFD. The data are presented as mean values ± SD. ^*^P < 0.05; ^**^P < 0.01; ^***^P < 0.001, Student’s *t-*test

To elucidate the physiological role of Grail in obesity development in vivo, we used a Grail KO mouse model to determine the effects of Grail on diet-induced obesity (Supplementary Fig. 2). To that end, the wild-type (WT) and Grail KO mice were fed normal diet (ND) or HFD for 16 weeks and their body weights were determined. We observed a significant decrease in the body weight and weight gain in the Grail KO mice fed ND (Grail KO, 26.5 g; WT: 31.1 g; Grail KO, 33%; WT, 44%, respectively) or HFD (Grail KO, 34.8 g; WT, 45.5 g; Grail KO, 78%; WT, 104%, respectively), compared with those determined in the WT mice (Fig. 5a–c). However, the food intake was shown to be similar for both WT and Grail KO mice fed either ND or HFD (Fig. 5d). The weights of the epididymal adipose tissue (EWT) and subcutaneous adipose tissue (SWT) were compared between the WT and Grail KO mice fed ND or HFD, and were shown to be lower in the Grail KO mice compared with those in the WT mice (Fig. 5e, f). We examined whether these differences in both ND and HFD-induced weight gain are related to the alterations in adiposity. Morphometric analysis revealed that the adipocytes from the ND- or HFD-fed Grail KO mice were smaller than the adipocytes isolated from the WT mice (Fig. 5g, h).

### HFD-induced pro-inflammatory cytokine expression is reduced in Grail KO mice

**Fig. 6:**
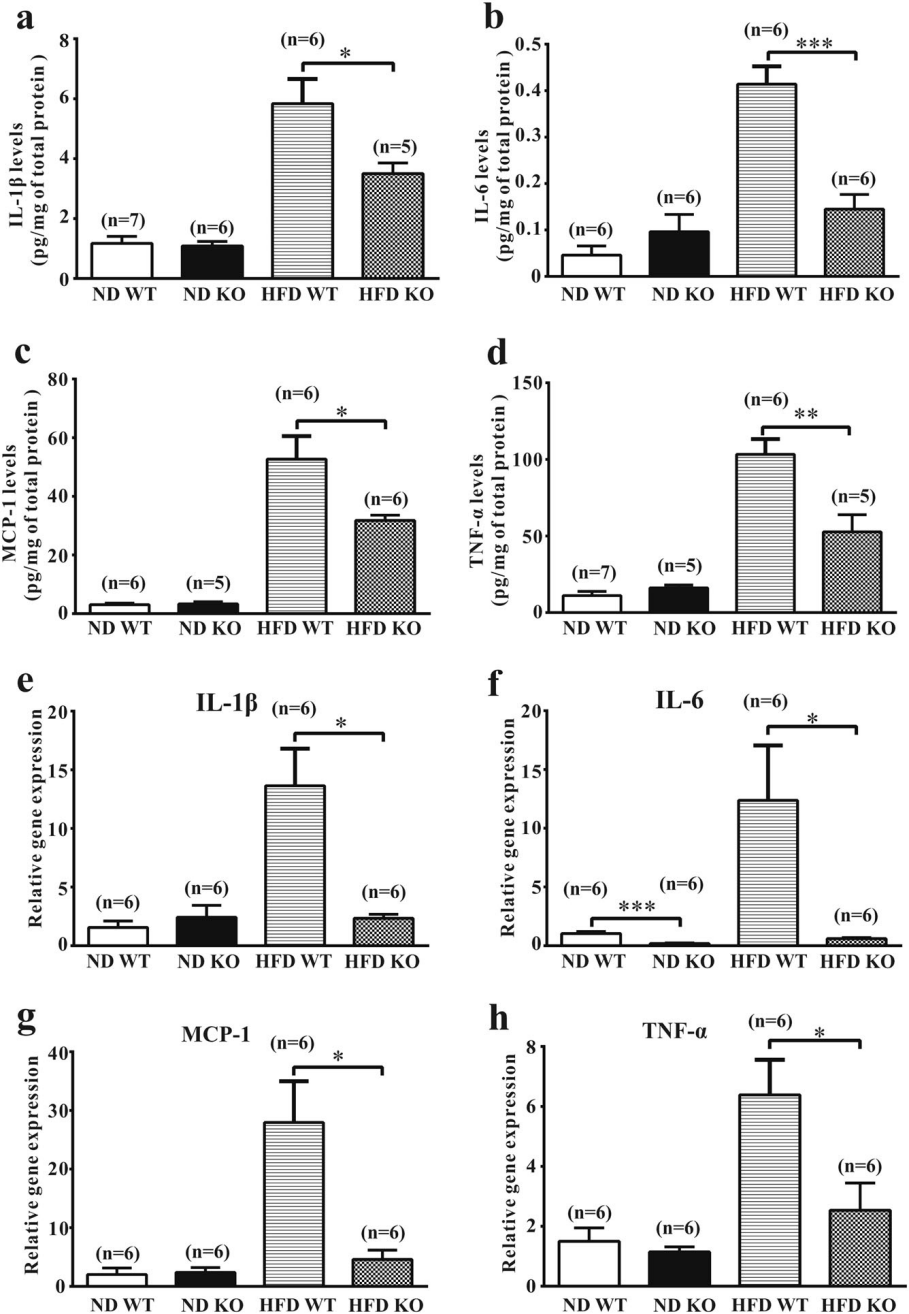
Pro-inflammatory cytokine expression levels are reduced in the HFD-fed Grail KO mice. **a**–**h** IL-1β, IL-6, MCP-1, and TNF-α mRNA and protein levels in the EWT obtained from the ND- or HFD-fed WT and Grail KO mice. The data are presented as mean values ± SD. ^*^P < 0.05; ^**^P < 0.01; ^***^P < 0.001, Student’s *t-*test

Obesity is associated with chronic low-grade inflammation in metabolic tissues, especially the adipose tissue, which may play a critical role in the pathophysiology of metabolic syndrome. Many cytokines, such as TNF-α, IL-6, IL-1β, and MCP-1, are involved in this inflammatory response^15^. We analyzed inflammatory cytokine levels in the adipose tissue samples isolated from the ND- and HFD-fed mice. TNF-α, IL-6, IL-1β, and MCP-1 mRNA and protein expression levels in the white adipose tissue of HFD-fed WT mice were shown to be significantly higher than those in the Grail KO mice (Fig. 6 and Supplementary Fig. 3). In contrast, the expression of these cytokines did not differ between in the ND-fed WT and Grail KO mice (Fig. 6 and Supplementary Fig. 3).

### Glucose and insulin tolerance are improved in HFD-fed Grail KO mice

**Fig. 7:**
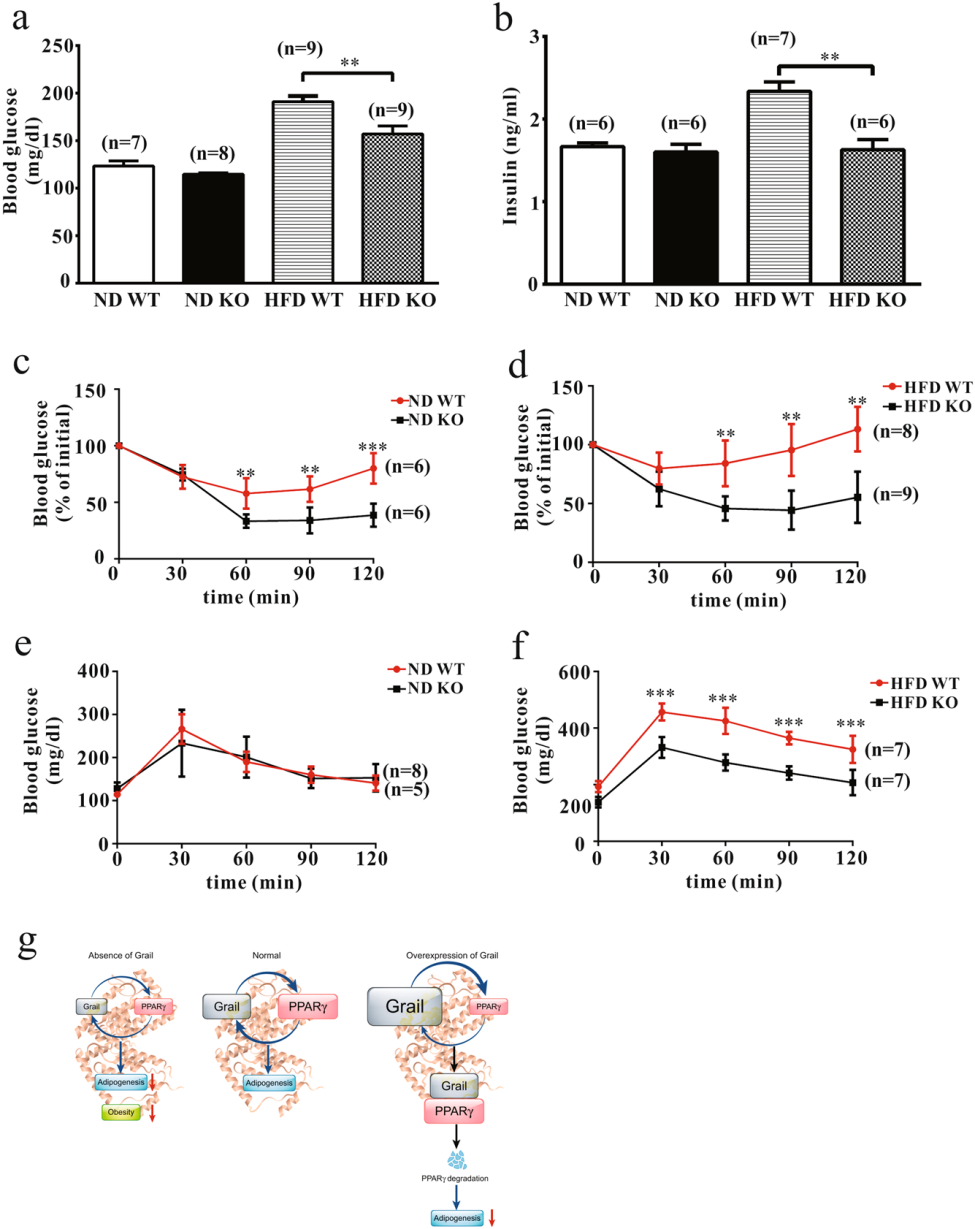
Glucose and insulin tolerance are improved in the Grail KO mice. **a**–**b** The fasting glucose and insulin serum levels were determined in the ND- and HFD-fed WT and Grail KO mice. **c**–**f** GTT and ITT results obtained using ND- and HFD-fed WT and Grail KO mice. **g** The proposed roles of Grail in adipogenesis and obesity. The inhibition of Grail expression leads to a decrease in the adipogenesis and diet-induced obesity. Grail overexpression suppresses PPARγ-mediated adipogenesis by targeting PPARγ for degradation. The data are presented as mean values ± SD. ^**^P < 0.01; ^***^P < 0.001, Student’s *t-*test

We further analyzed serum glucose and insulin levels. Glucose and insulin levels in the sera of HFD-fed Grail KO mice were shown to be significantly lower than those in the sera of the control mice (Fig. 7a, b). Furthermore, glucose tolerance test (GTT) and insulin tolerance test (ITT) were administered to the ND- and HFD-fed mice at 16 weeks to determine the effects of Grail KO on insulin and glucose homeostasis. ITTs showed that compared with that in the WT mice, ND- and HFD-fed Grail KO mice demonstrated hypoglycaemic responses (Fig. 7c, d). The GTT results showed that the blood glucose levels following the glucose injection were lower in the HFD-fed Grail KO mice than those in the HFD-fed WT mice (Fig. 7e, f), suggesting an improved glucose tolerance in the HFD-fed Grail KO mice.

## Discussion

Here, we demonstrated for the first time that Grail interacts with PPARγ, the master adipogenic factor, modulating its adipogenic activity, and that it is involved in adipocyte differentiation. The inhibition of Grail expression was shown to attenuate adipogenesis and adipocyte differentiation by reducing PPARγ expression, whereas Grail overexpression suppresses PPARγ-mediated adipogenesis as well, by targeting PPARγ for degradation (Fig. 7g).

Similarly, we observed that PPARγ expression is reduced in the adipose tissue of Grail KO mice (Supplementary Fig. 4). Furthermore, Grail expression was shown to be downregulated in the PPARγ-silenced cells. Based on the obtained results, we propose here that Grail represents a newly-identified regulator of adipogenesis and diet-induced obesity.

In this study, we demonstrated that Grail affects adipogenesis mainly through regulating PPARγ. Interestingly, the adipogenesis effect with Grail knockdown or PPARγ and Grail double knockdown differed from that noted in cells with only PPARγ knockdown. Recently, an increasing number of studies indicate that E3 ligases, such as MKRN1, CRL4, DTX4, NEDD4 and Fbxw7, play an important role in adipogenesis by regulating PPARγ or other major transcription factors^16–19^. However, whether these E3 ligases exhibit connections or interactions or even form networks during adipogenesis remains unknown. Grail not only alters PPARγ stability but also influences other factors in the differentiation process.

The intracellular level and activity of PPARγ are tightly regulated through post-translational modifications and allosteric modifications^20–22^. Here, we showed that Grail affects PPARγ expression and activity. As Grail is expressed in multiple tissues, it may affect PPARγ functions in other tissues, or be involved in the regulation of nuclear receptors involved in metabolic homeostasis, which should be determined in further studies.

Our in vivo results demonstrated that Grail KO mice have lower body weights and gain less weight than the WT mice when fed both ND and HFD, which was not caused by the differences in food intake. This suggests that Grail KO mice show the resistance to obesity development. The epididymal and subcutaneous adipose fat pads of Grail KO mice were shown to weigh significantly less than those in the control mice, and that the adipocyte in the in the EWT and SWT of ND- or HFD-fed Grail KO mice were much smaller than those in the controls. In addition, the expression of PPARγ was significantly reduced in the adipose tissue of ND- or HFD-fed Grail KO mice, which supports our conclusions. Increasing evidence from in vitro and in vivo studies suggests that the brown and beige adipose tissues increase whole-body energy expenditure through nonshivering thermogenesis and therefore can protect against obesity and diabetes^23,24^. PPARγ is characterized as a major regulator of white and brown adipocyte differentiation^25,6^. Our study demonstrates that Grail can affect white adipocyte differentiation by mediating PPARγ expression and function. Based on this evidence, investigating the effect of Grail in the regulation of energy homeostasis and brown adipocyte differentiation would be of potential interest.

Obesity is associated with chronic low level inflammatory processes in the metabolic tissues, especially the adipose tissue. Many cytokines are involved in this response^26^. Recently, Grail was reported to exert a crucial role in the control of T-cell tolerance and immune response^27^. However, the role of Grail in adipose tissue inflammation has not been characterized. Here, we showed that the expression of pro-inflammatory cytokines, such as TNF-α, IL-6, IL-1β, and MCP-1, is upregulated in the adipose tissues of HFD-fed WT mice but not in the Grail KO mice. Taken together, these data suggest that HFD-induced adipose tissue inflammation is reduced in Grail KO mice. Obese adipose tissue also exhibits marked macrophages infiltration. These macrophages secrete TNF-α via nuclear factor (NF)-κB signaling. Macrophage-secreted TNF-α can induce FFA secretion via activating the TNFR signaling pathway in adipocytes. FFAs serve as naturally occurring ligands for Toll-like receptor 4 to promote macrophage activation by inducing the NF-κB signaling pathway. Therefore, adipocytes and macrophages can form a vicious cycle to augment inflammatory responses via a paracrine loop involving TNF-α and FFAs^28–30^. Recently, evidence has also demonstrated that Grail is involved in the polarization of adipose tissue macrophages^31^. However, the detailed mechanism underlying the role of Grail in the adipocyte and macrophage interaction remains unclear. It will be interesting to explore whether Grail modulates the paracrine loop between adipocytes and macrophages.

Furthermore, HFD-induced glucose and insulin tolerance were shown to be less severe in the Grail KO mice than in WT mice, which is consistent with the previous results showing that the induction of pro-inflammatory cytokines in adipose tissue in obesity can lead to the development of metabolic syndrome and insulin resistance^32^. Therefore, our results suggest that Grail may be involved in adipose tissue inflammation.

p53 was a multi-functional tumor suppressor involved in cellular responses to various stresses, including DNA damage, hypoxia, and oncogene expression. Activation of p53-induced cell responses such as cell cycle arrest, senescence, and apoptosis, which contribute to its function in tumor suppression either by maintaining genomic integrity or eliminating potentially oncogenic cells through apoptosis^33–36^. Recent studies have demonstrated that the intake of excessive amounts of nutrients causes p53-induced inflammation in adipose tissue, leading to insulin resistance and diabetes in mice^37^. Moreover, p53 activation affects the white and brown adipocyte differentiation and diet-induced obesity^38,39^. Based on our results, Grail affects adipogenesis either by enhancing PPARγ gene expression or targeting it for degradation. However, p53 expression in Grail-mediated adipogenesis is uncharacterized. This is an interesting issue, and we should further dissect the potential correlation between Grail and p53 in adipocyte differentiation and diet-induced obesity. In conclusion, our findings provide new insight into the role of Grail in adipogenesis and obesity development.

## Methods

### Animals experiments

All animal experiments were approved by the National Defense Medical Center Animal Experiment Ethics Committee (IACUC-16-028). The Grail KO mice were generated by the Transgenic Mouse Models Core (Taipei, Taiwan) using CRISPR-Cas9 technology, which induced an NHEJ-mediated deletion in Grail exon 1, resulting in the removal of the first start codon, and the KO mice were generated on a C57BL/6 J background. The mice were housed under a regular 12-h light/dark cycle for 2 weeks before the initiation of the experiments. Six-week-old male WT and Grail KO mice were fed ad libitum with either a ND or HFD D12451 (Research Diets, New Brunswick, NJ) for 16 weeks. The mice were kept on a 12 h light/dark cycle at 22 ± 1°C.

### Cells, plasmids, and transfection procedures

Preadipocyte 3T3-L1 cells were cultured in Dulbecco’s modified Eagle’s medium (DMEM) supplemented with 10% fetal bovine serum. To induce the differentiation, 3T3-L1 cells were grown to confluence in 10% calf serum/DMEM and treated with isobutyl methylxanthine, dexamethasone, and insulin. The Grail gene was cloned into the pCMV-TNT vector (Promega, CA, USA) using the *Eco*RI and *Bam*HI sites. The pcDNA-flag-PPARγ plasmid was purchased from Addgene. Transfection was performed using Fugene 6 (Roche, Basel, Switzerland), according to the manufacturer’s instructions. Cells were plated at a low density (~ 1×10^5^ cells/60-mm dish) and allowed to grow to 30–40% confluence, which was followed by Fugene 6-mediated gene transfection. The transfected cells were harvested at 48 h and lysed in the radioimmunoprecipitation buffer (100 mM Tris-HCl pH 8.0, 150 mM NaCl, 0.1% SDS, and 1% Triton x-100).

### Immunoprecipitation and immunoblotting

Cells were harvested in the lysis buffer (50 mM Tris pH 8.0, 5 mM NaCl, 0.5% NP-40, and 1 × protease inhibitor), freeze/thawed three times, and the proteins were recovered. Protein concentration was determined using the Bradford method (Bio-Rad, CA, USA). Cell extracts containing equivalent amounts of protein were immunoprecipitated overnight at 4°C in lysis buffer containing a polyclonal antibody against Grail or PPARγ. Dynabeads Protein G (Invitrogen) was added to the immunoprecipitation mixture for 1 h. Afterward, the samples were washed three times with SNNTE buffer (5% sucrose, 1% NP-40, 0.5 M NaCl, 50 mM Tris, pH 7.4, and 5 mM EDTA). The immunoprecipitates were resuspended in sodium dodecyl sulfate polyacrylamide gel electrophoresis (SDS-PAGE) sample buffer, boiled, and loaded onto an SDS-PAGE. The separated proteins were transferred to a nitrocellulose membrane and the blot was probed with the indicated primary antibodies followed by a secondary antibody (horseradish peroxidase-conjugated anti-mouse or anti-rabbit IgG in phosphate-buffered saline/Tween 20 with 5% Carnation non-fat milk). Proteins of interest were detected using enhanced chemiluminescence reagents (GE Healthcare). The primary antibodies used for immunoblotting were: anti-PPARγ (81B8, Cell Signaling, USA), anti-actin (MAb1501, Chemicon, USA), and anti-Grail antibodies.

### Virus particle production, viral transduction, and RNA interference

Grail was cloned into the retroviral plasmid vector pQCXIP (Clontech). The pQCXIP-Grail and pQCXIP-empty plasmids were transfected into GP2-293 cells using Fugene 6. Grail retroviruses were prepared according to the protocol published on the Clontech website. The shRNA oligonucleotides were cloned into the retroviral siRNA expression vector, pSIREN-Retro-Q (Clontech). Retroviruses overexpressing Grail shRNA were generated according to the protocol published on the Clontech website. (Grail shRNA target sequence 1: 5′-gaggcatccaagtcacaatgg-3′; Grail shRNA target sequence 2: 5′-gcaggaagcagaggcagttaa-3′). Cells were infected with the indicated retroviruses in the selection medium containing 2 μg/mL polybrene. Forty-eight hours after the infection, the cells were treated with 8 μg/mL puromycin to select for the pool of puromycin-resistant clones. The AAV expression vector was constructed and manipulated using the Helper Free shRNA Expression System (Cell Biolabs). The shRNA oligonucleotides were cloned into the AAV shRNA expression vector pAAV-U6-GFP. AAVs overexpressing PPARγ shRNA were generated according to standard protocols. (PPARγ shRNA target sequence 1: 5′-gccctggcaaagcatttgt-3′; PPARγ shRNA target sequence 2: 5′-gccctttaccacagttgat-3′).

### Real-time quantitative PCR

RNAs from cells and tissues were isolated using the TRIzol reagent (Sigma-Aldrich, St. Louis, MO, USA). Complementary DNAs were synthesized using Epicentre MMLV. Cellular gene expression was analysed using an Applied Biosystems 7500 Real-Time PCR system and the IQ2 FAST Q-PCR kit. Gene expression in the tissues was determined using a Roche LightCycler 480. The primers used are listed in Supplementary Table 1.

### Immunohistochemistry and Oil Red O staining

Adipose tissue was fixed overnight in 4% formaldehyde at room temperature, and then embedded in paraffin. The sections were mounted on glass slides, deparaffinised using xylene and stained with haematoxylin–eosin. For Oil Red O staining, the cells were fixed in 10% formaldehyde, washed twice with 60% isopropanol and stained with Oli Red O. The absorbance was measured at 510 nm.

### GTT

After 16 weeks, Grail KO and WT mice fasted overnight and were administrated intraperitoneally with 1 g/kg body weight of glucose. Following this, blood samples were collected from the tail and used to determine glucose concentrations (Accu-Chek Mobile, Roche).

### ITT

Grail KO and WT C57BL/6 J mice fasted for 6 h after 16 weeks of ND or HFD administration, and the mice were injected intraperitoneally with 1 IU/kg insulin. Following this, blood samples were collected from the tail used to determine the glucose concentration on a Roche Accu-Chek Mobile.

### Insulin, IL-1β, IL-6, TNF-α, and MCP-1 levels

The expression of adipokines and cytokines (insulin, IL-1β, IL-6, TNF-α, and MCP-1) in serum and tissue lysates was determined using the Bio-Plex Multiplex Immunoassay kit (Bio-Rad), according to the manufacturer instructions. Blank values were subtracted from all readings. All assays were performed directly at room temperature in a 96-well round-bottomed microtiter plates (Muller Ratiolab, Dreieich, Germany) protected from light. Measurements and data analysis were performed with a Bio-Plex system in combination with Bio-Plex Manager software.

### Statistical analysis

Student’s two-tailed *t* -test was used to determine differences between two groups. *P* *<* 0.05 was considered statistically significant.

## Electronic supplementary material

Supplementary Information
