# Supplementary material for: Grail is involved in adipocyte differentiation and diet-induced obesity
Source: Cell Death Dis. 2018 May 9;9(5):525. doi: 10.1038/s41419-018-0596-8 (PMC5943410; doi:10.1038/s41419-018-0596-8)
Supplement: Supplementary file 1 — Supplementary Information [file 41419_2018_596_MOESM1_ESM.docx]

**Supplementary Information**

**
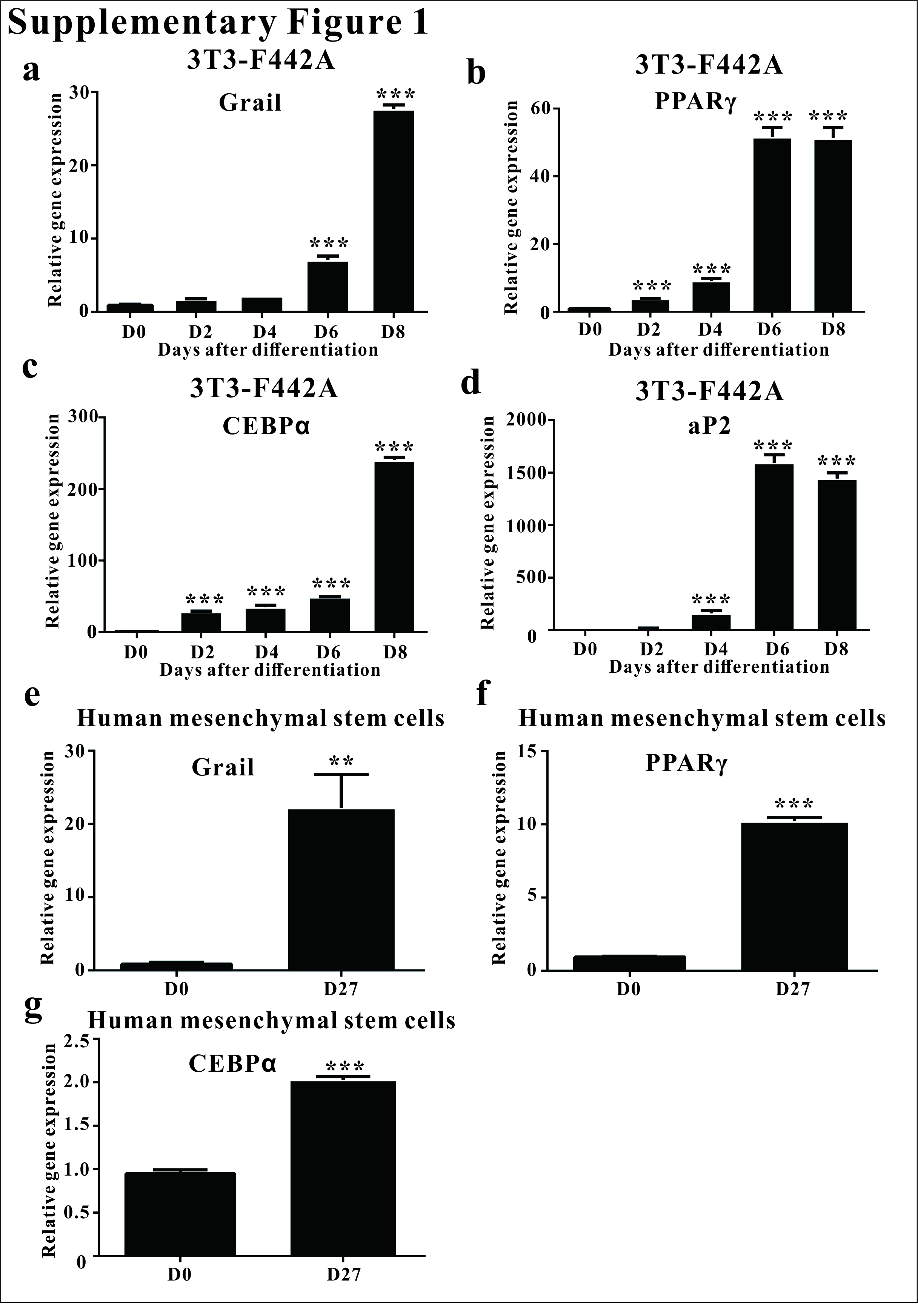
**

**Supplementary Fig. 1** Grail expression is induced in 3T3-F442A and human mesenchymal stem cells during adipocyte differentiation. (a-g) Grail mRNA expression during adipogenesis. PPARγ, C/EBPα and aP2 were analysed as adipogenic markers.

**
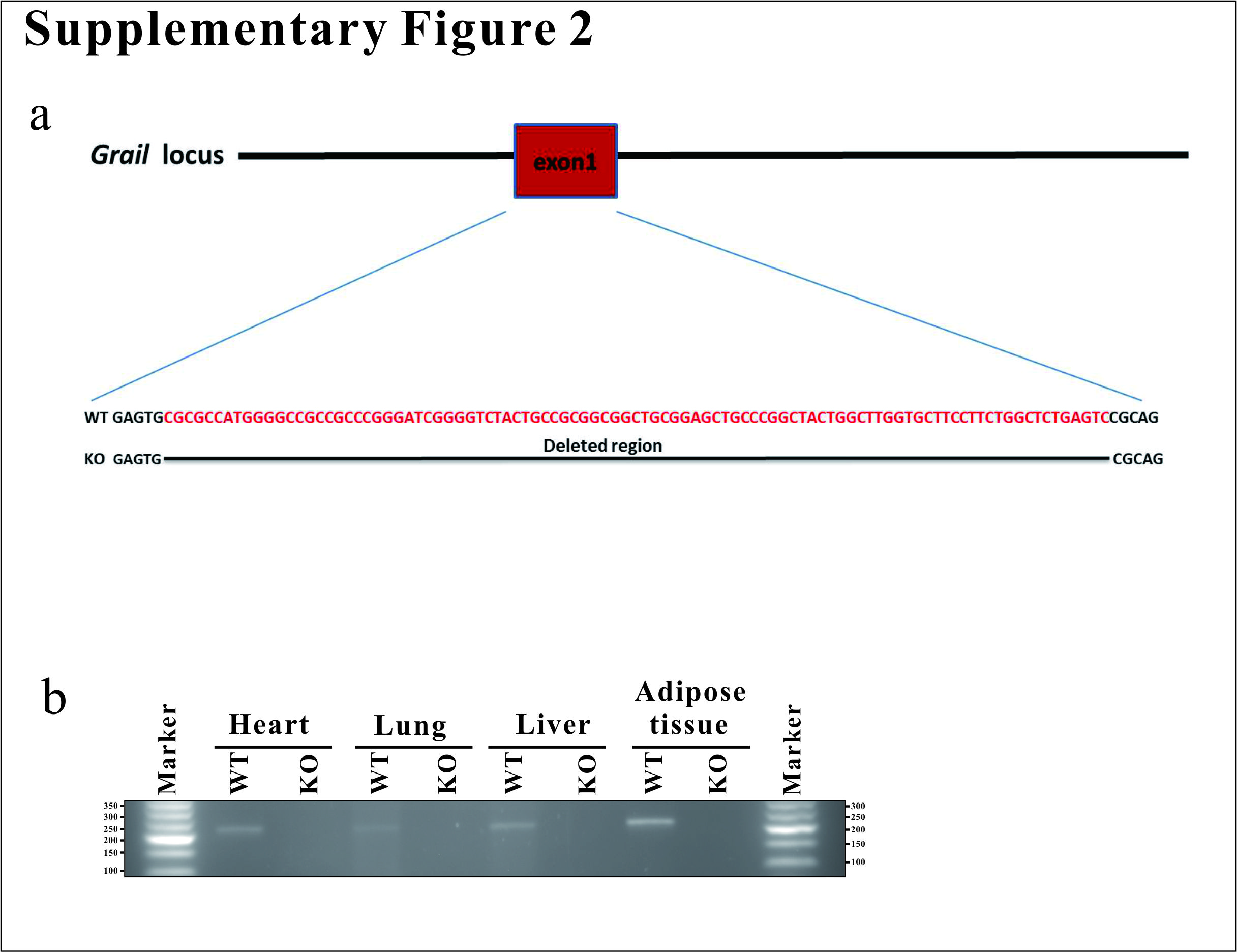
**

**Supplementary Fig. 2** Grail KO mouse generation. (a) Schematic representation of the targeted mouse Grail exon 1, and the WT (upper) and Grail KO (lower) sequence. (b) Genotyping PCR for Grail expression in the indicated organs of the Grail KO and WT mice.

**
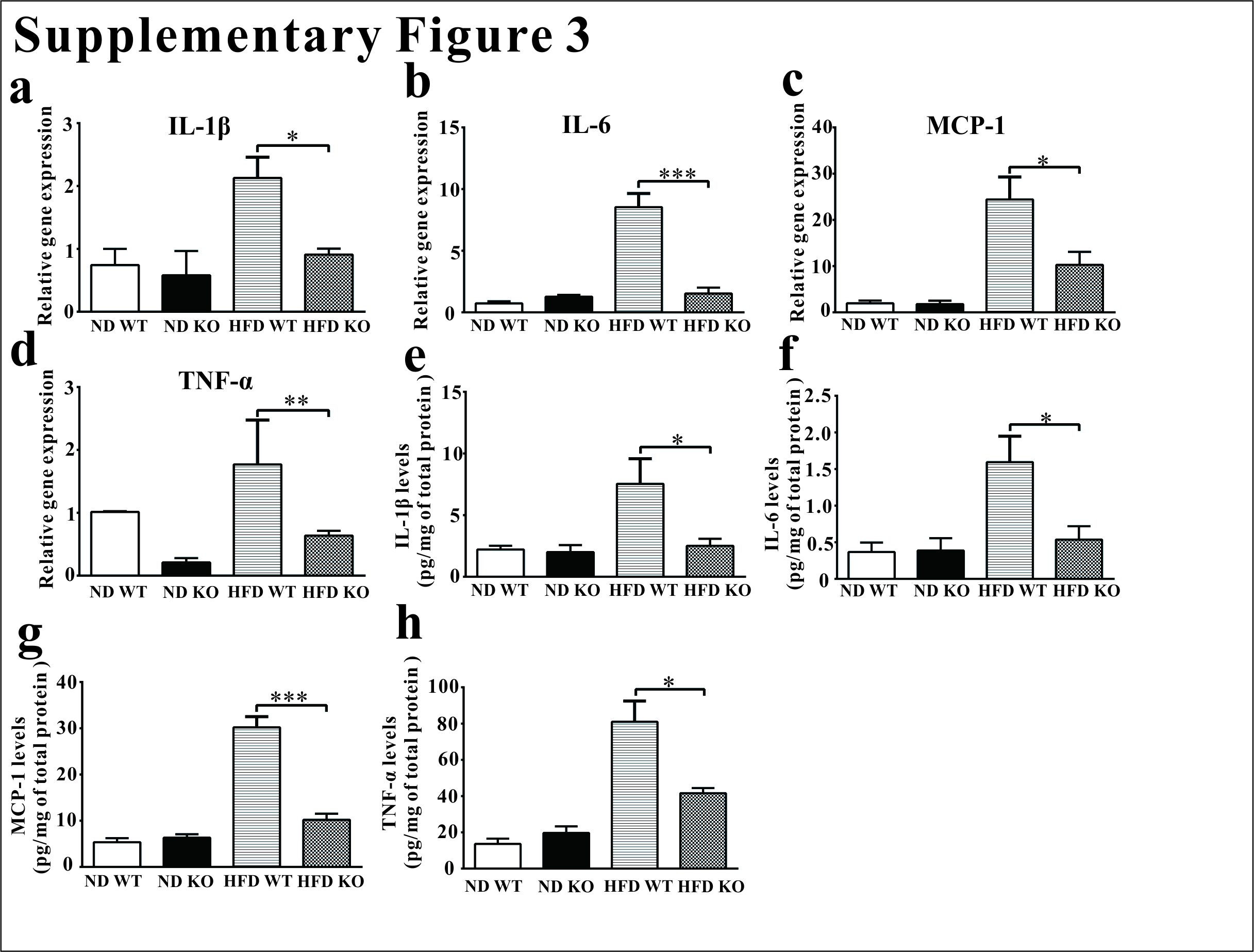
**

**Supplementary Fig. 3** Proinflammatory cytokine expression levels are decreased in the HFD-fed Grail KO mice. (a-h) IL-1β, IL-6, MCP-1 and TNF-α mRNA and protein levels were determined in SWT obtained from the ND- or HFD-fed WT and Grail KO mice. The data are presented as mean values ± SD (n=5–7). ^*^*P*<0.05; ^**^*P*<0.01; ^***^*P*<0.001, Student’s *t-*test

**
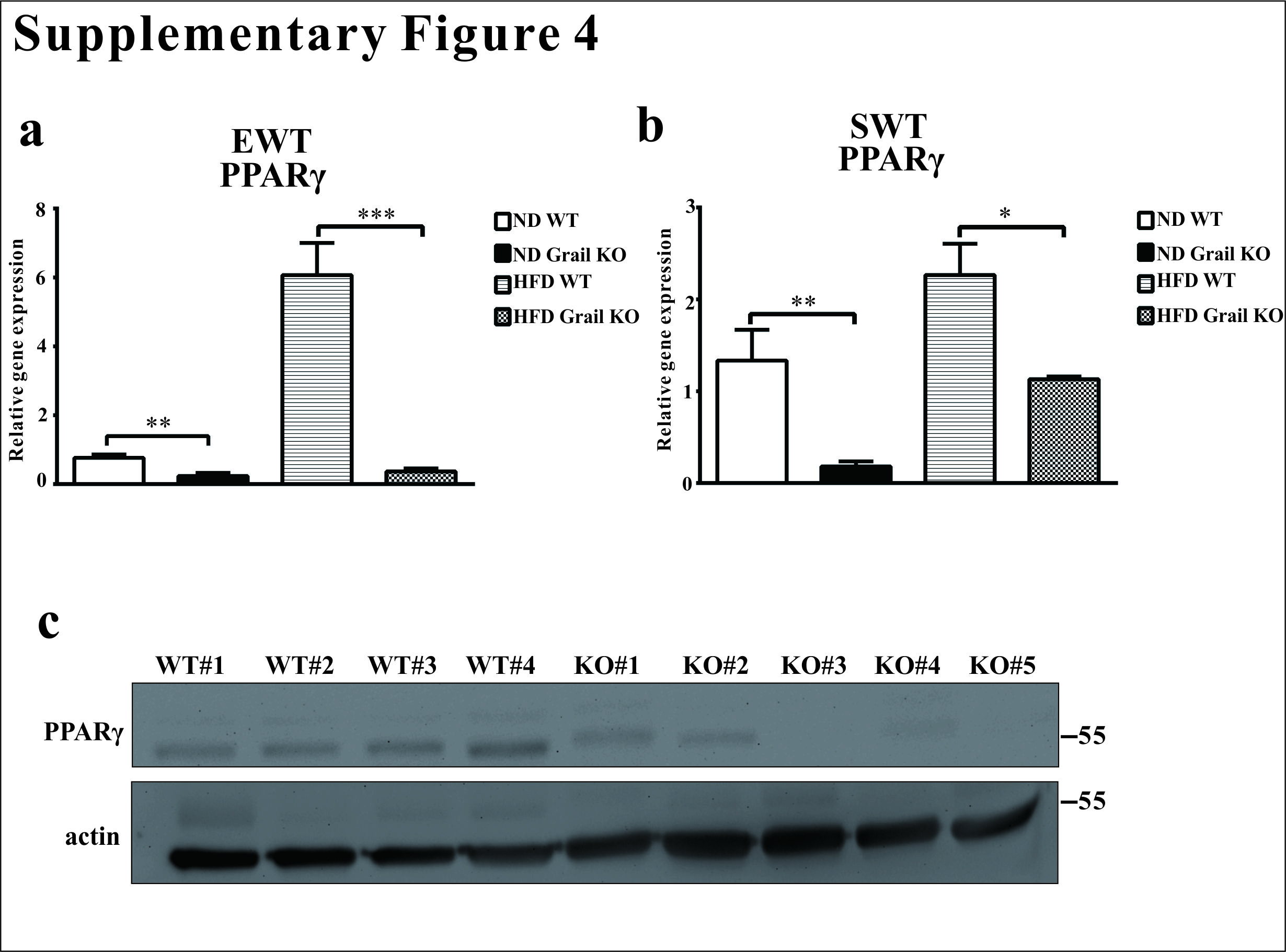
**

**Supplementary Fig. 4** The expression of PPARγ is reduced in the adipose tissues of the Grail KO mice. (a-b) PPARγ mRNA levels in the EWT and SWT samples obtained from the HD- and HFD-fed WT and Grail KO mice. (c) PPARγ protein levels in the EWT samples obtained from WT and Grail KO mice. The data are presented as mean values ± SD (n=5–7). ^*^*P*<0.05; ^**^*P*<0.01; ^***^*P*<0.001, Student’s *t-*test


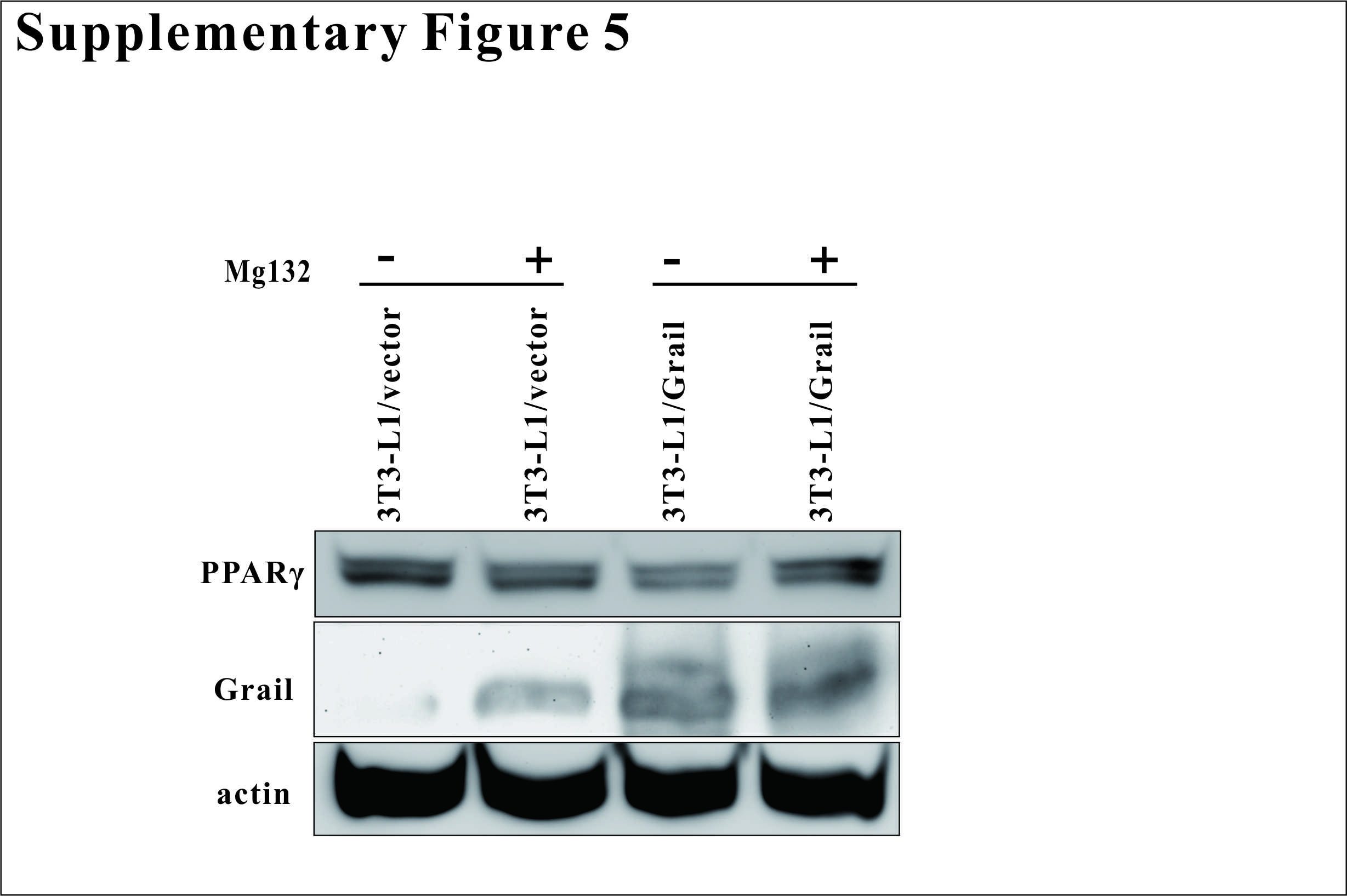


**Supplementary Fig. 5** Grail reduces PPARγ protein levels. The 3T3-L1/vector and 3T3-L1/Grail cells were transfected with 0.5 μg of pcDNA-flag-PPARγ in the presence or absence of Mg132. The cells were then harvested and subjected to western blotting.

| **Supplementary Table 1 \| Primers used for genotyping and Q-PCR analysis** | | |
| --- | --- | --- |
| **Primers** | **Forward sequence** | **Reverse sequence** |
| **Genotyping** | | |
| **Grail KO** | *5’-cttgcttgtaggagctgcgtct-3’* | *5’-ctcagagccagaaggaagc-3’* |
| **Q-PCR for cell lines** | | |
| **Grail** | *5’-gcgtctggagccgtcatcttta-3’* | *5’-gggccatgttttttccctacttctat-3’* |
| **PPARγ** | *5’-gagctgacccaatggttgctg-3’* | *5’-gcttcaatcggatggttcttc-3’* |
| **C/EBPα** | *5’-gaacagcaacgagtaccgggta-3’* | *5’-gccatggcccttgaccaaggag-3’* |
| **aP2** | *5’-gaacctggaagcttgtcgcc-3’* | *5’-accagcttgtcaccatctcg-3’* |
| **actin** | *5’-gtggggcgccccaggcacca-3’* | *5’-ctccttaatgtcacgcacgatttc-3’* |
| **Q-PCR for adipose tissue** | | |
| **Grail** | *5’-aaatgcaagagctcaaagcag-3’* | *5’-gcagctgaagctttccaatag-3’* |
| **PPARγ** | *5’-ttatagctgtcattattctcagtggag-3’* | *5’-gactctgggtgattcagcttg-3’* |
| **C/EBPα** | *5’-aaacaacgcaacgtggaga-3’* | *5’-gcggtcattgtcactggtc-3’* |
| **IL-1β** | *5’-tgagcaccttcttttccttca-3’* | *5’-gcagctgtctaatgggaacg-3’* |
| **IL-6** | *5’-tctaattcatatcttcaaccaaga-3’* | *5’-tggtccttagccactccttc-3’* |
| **MCP-1** | *5’-ggctggagagctacaagagg-3’* | *5’-ctcttgagcttggtgacaaaaa-3’* |
| **TNF-α** | *5’-cgagtgacaagcctgtagcc-3’* | *5’-ttgagatccatgccgttg-3’* |
| **actin** | *5’-ctaaggccaaccgtgaaaag-3’* | *5’-accagaggcatacagggaca-3’* |
